# A narrative review of phase III and IV clinical trials for the pharmacological treatment of Huntington’s disease in adults

**DOI:** 10.1097/MD.0000000000041073

**Published:** 2024-12-27

**Authors:** Mohammed S. Alharthi

**Affiliations:** a Department of Clinical Pharmacy, College of Pharmacy, Taif University, Taif, Saudi Arabia.

**Keywords:** clinical trials, genetic, Huntington disease, neurodegenerative disorder, phase III and IV

## Abstract

Huntington disease (HD) is a hereditary neurodegenerative condition characterized by progression of motor, cognitive, and psychiatric abnormalities. Despite the lack of definitive medications, current research efforts are actively assessing novel pharmaceutical interventions through phase III and IV clinical trials to mitigate the limited effectiveness of existing therapeutic approaches. The primary objective of these trials is to enhance symptom management and improve the overall quality of life for individuals diagnosed with HD. These trials show potential for development of further efficacious therapeutic interventions in future. To identify and provide details about medications tested in completed phase III and IV clinical trials for managing HD in adults. Publicly available and relevant phase III and IV trials registered at ClinicalTrials.gov analyzed. Usage of the trialed medications for HD reviewed. As of November 10, 2023, there were 242 phase III and IV clinical trials related to HD. Eight clinical trials from these met the inclusion criteria for the current study. The medications used in phase III and IV trials are minocycline, valbenazine, deutetrabenazine, tominersen, pridopidine (phase III), and memantine (phase IV). Evaluating phase III and IV clinical studies on HD highlights the importance of tailored approaches for each patient’s unique disease presentation. Current medications aim to manage HD symptoms, potentially improving outcomes and reducing disease progression risks. The growing emphasis on specific approaches reflects a better understanding of HD’s diverse symptoms, presenting opportunities for more effective and personalized treatment strategies.

## 1. Introduction

Huntington disease (HD) is a genetic neurodegenerative disorder characterized by progressive neuronal degeneration in the brain.^[1]^ The etiology of this illness can be attributed to a genetic mutation that results in the synthesis of an atypical variant of the huntingtin protein.^[2]^ It manifests with various motor, cognitive, and mental symptoms.^[3]^ The pathogenesis of HD is characterized by the progressive deterioration of specific regions of the brain, especially the striatum. This degeneration process results in an imbalance in neurotransmitters, which affects the transmission of signals between neurons.^[4]^ HD is characterized by a slow and steady decline of neurons, leading to a gradual deterioration of motor and cognitive abilities over time.^[5]^ The involuntary spasmodic or convulsive movements notably affect an individual’s motor coordination and equilibrium.^[6]^ Cognitive impairment is a notable characteristic, resulting in challenges related to attention, memory deficits, and compromised cognitive processes involved in decision-making.^[7]^ Psychiatric symptoms like depression, anxiety, and irritability complicate the clinical presentation of HD.^[8]^ The chance of inheriting HD from an affected parent is 50% due to its hereditary nature.^[9]^ Mutant huntingtin (mHtt) triggers the death of neurons in the striatum by initiating a series of biochemical processes. At the outset, it causes disturbances in essential signaling pathways implicated in neurotransmission and synaptic plasticity, compromising the communication between neurons. Simultaneously, mHtt disrupts the functioning of mitochondria, resulting in reduced energy generation and increased oxidative stress, further compromising the integrity of cells. In addition, mHtt facilitates the creation of harmful protein clusters by causing abnormal interactions between proteins, which disrupts cellular functions and encourages neural dysfunction. The complex interaction of molecular dysregulation eventually leads to the death of neurons in the striatum, which is a significant characteristic in the development of HD.^[10]^ mHtt in HD causes neural circuit anomalies in the cortico-striatal-thalamo-cortical circuit, which controls motor control and cognition. The striatum degenerates due to mHtt-induced neurotransmission abnormalities, neuronal excitability, and excitotoxicity. Disorders in connected brain regions aggravate motor and cognitive deficiencies. Neurodegeneration spreads as the condition progresses, worsening symptoms.^[11]^ Neuroinflammation is a significant factor in the neurodegenerative progression of HD, with disturbances in neuronal circuits and the buildup of mHtt aggregates. Increased concentrations of pro-inflammatory cytokines and activated glial cells have a role in impairing neurons and worsening disease progression. Approaches aimed at addressing neuroinflammation, such as utilizing minocycline, an antibiotic with anti-inflammatory characteristics, have shown potential in preclinical investigations via the mitigation of microglial activation and the reduction of neuronal degeneration. Moreover, neurodegeneration mediated by mHtt entails the activation of apoptotic pathways, resulting in the deliberate demise of damaged neurons.^[12]^ Individuals’ disease progression varies and is impacted by variables such as mHtt accumulation. Those with more CAG repeats may rapidly deteriorate motor and cognitive function.^[13]^

The symptoms of this condition typically manifest during middle adulthood, with variations in intensity and timing among individuals.^[14]^ Although a definitive treatment for HD is not yet available, the medical community focuses on implementing supportive therapies and administering medications to effectively address symptoms and enhance the overall well-being of persons affected by the condition.^[15–17]^ A meta-analysis of 4 randomized controlled trials with 1130 patients (816 receiving pridopidine, 314 receiving placebo) found a nonsignificant trend favoring pridopidine in the Unified HD Rating Scale Total Motor Score (mean difference, −0.93; 95% CI, −2.01 to 0.14; *P* = .09). In 3 investigations, pridopidine significantly improved the Unified HD Rating Scale Modified Motor Score (mean difference, −0.81; 95% CI, −1.48 to −0.13; *P* = .02) compared to placebo. Pridopidine was well-tolerated, with no significant increase in adverse events (relative risk, 1.03; 95% CI, 0.94–1.13; *P* = .49) or major adverse events (relative risk, 1.62; 95%, 0.88–2.99; *P* = .12).^[18]^ Various medications are available to effectively reduce symptoms and improve the overall well-being of people affected by the condition. Tetrabenazine is effective in reducing chorea and providing relief from involuntary motor disorders. Additionally, psychotropic medications like risperidone or olanzapine can be prescribed to manage mental symptoms effectively.^[17,19]^

Changes significantly influence the presentation of symptoms in HD in dopamine levels. The process of striatal degeneration, characterized by a high concentration of dopamine receptors, leads to the disruption of dopaminergic signaling. The depletion of medium spiny neurons is associated with a reduction in the suppression of dopamine release, leading to the dysregulation of dopamine levels. The imbalance of motor function and psychological symptoms is often reported in individuals with HD.^[20]^ Excessive release of glutamate and inadequate GABA signaling lead to excitotoxicity and neuronal damage. Impaired glutamate and dopamine signaling exacerbate neuronal dysfunction and exacerbate symptoms of HD. Therefore, treating HD may include addressing glutamate dysregulation.^[21]^ In addition to motor symptoms, individuals with HD frequently experience psychiatric manifestations, including mood disturbances and anxiety.^[22]^ Antidepressant medications, specifically selective serotonin reuptake inhibitors and anxiolytics are commonly prescribed to target the various manifestations of the disease’s clinical presentation.^[23]^ Clonazepam, which belongs to the benzodiazepine class of medications, is utilized as an additional pharmacological intervention for the management of HD.^[24]^ The utilization of this medication extends to the mitigation of chorea and its related symptoms using its modulation of GABAergic neurotransmission.^[25]^

Although several studies have investigated the potential neuroprotective effects of coenzyme Q10, an antioxidant, and creatine supplementation in HD, the available evidence is inconclusive.^[26,27]^ Therefore, additional research is needed to determine the effectiveness and safety of these interventions. As scientific research progresses, the field of pharmacological interventions for HD may change. This review covers several medications used to manage symptoms of HD, including minocycline, valbenazine, deutetrabenazine, Tominersen, pridopidine (phase III), and memantine (phase IV). This review aims to provide a comprehensive overview of the current pharmacological treatments evaluated in phase III and IV clinical trials for HD. By analyzing completed trials, this paper seeks to identify the most promising therapeutic options and their mechanisms of action, assess their efficacy and safety, and highlight areas where further research is needed.

## 2. Methods

### 2.1. Data sources

ClinicalTrials.gov served as the primary data source for this review. ClinicalTrials.gov is a comprehensive public registry encompassing clinical trials conducted across 221 countries. It catalogues medical studies involving human volunteers and includes information on interventional studies. The search was conducted from inception until November 10, 2023, retrieving data from 242 relevant studies. Keywords utilized for the search included “Huntington’s,” “Huntingtin gene,” and “Chorea.” Inclusion criteria were defined as follows:

- Primary focus on HD.
- Phase III and IV interventions.
- Completion of trials.
- Inclusion of adult patients (age range: 18–64 years).
- Availability of results.
- Interventional studies.

**Figure 1.**
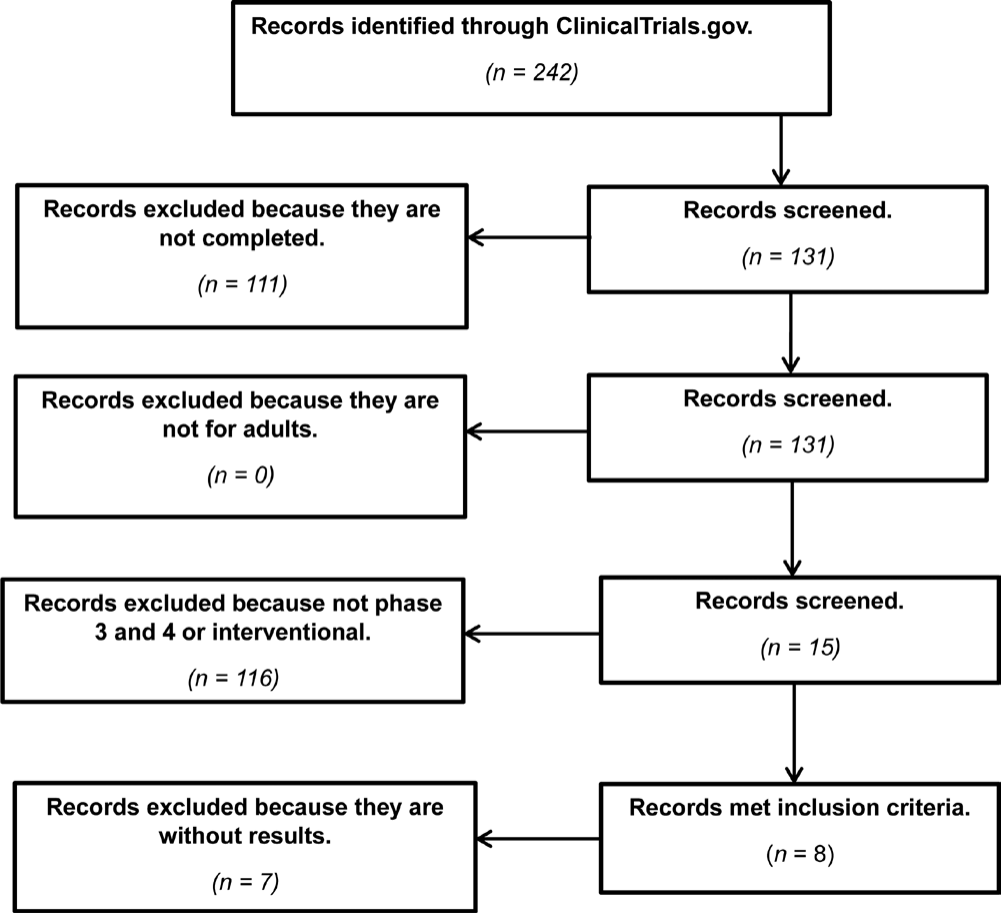
Summary of review process.

The review process has been summarized in Figure 1.

### 2.2. Data collection and coding

A systematic approach was employed for data collection and coding. For each study, key parameters were documented, including study status (e.g., recruitment status, completion status), availability of results, eligible populations (e.g., gender, age groups), and study type (observational or intervention-based). Additionally, study phases were identified and recorded. Information on each investigated drug and the geographical locations of all studies were documented. Following data collection, all information was coded manually to ensure accuracy and consistency in categorization and analysis. This coding facilitated the organization and interpretation of data for subsequent analysis.

### 2.3. Study design selection

Trials were selected for inclusion based on predetermined criteria. Specifically, trials involving non-phase III and IV treatments were excluded. The selection process ensured a focused analysis of HD management interventions.

## 3. Results

Eight trials were included in this review after excluding trials. The medications used in phases III and IV are minocycline, valbenazine, deutetrabenazine, tominersen, pridopidine (phase III), and memantine (phase IV). The title of the trial, location, medication intervention, dose/regimen, route of administration, comparison, primary outcome measures, and phase are shown in Table 1.

**Table 1:** Data from http://clinicaltrials.gov as of November 10, 2023.

Study title	Location	Intervention (medication)	Route of administration	Comparison	Primary outcome measures	Phase
Pilot Study of Minocycline in Huntington Disease	United states, Canada	Minocycline	Oral	Placebo	• Change from baseline to month 18 in the total functional capacity (TFC) scale. • Establish a preliminary estimate of minocycline’s impact on the progression of HD. • Assess the futility of further study of minocycline.	Phase II and III
First-Time Use of SD-809 in Huntington Disease	United states, Australia, Canada	SD-80	Oral	Placebo	Change “From Screening Period Baseline” to “During Maintenance Period” in the Unified HD Rating Scale.	Phase III
Efficacy, Safety, and Tolerability of Valbenazine for the Treatment of Chorea Associated with Huntington Disease	United states, Canada	Valbenazine	Oral	Placebo	Change “From Screening Period Baseline” to “During Maintenance Period” in the unified HD Rating Scale. (UHDRS).	Phase III
A Study to Evaluate the Efficacy and Safety of Intrathecally Administered RO7234292 (RG6042) in Participants with Manifest Huntington Disease	United states, Argentina, Australia, Austria, Canada, Chile, Denmark, France, Germany, Italy, Japan, Netherland, New Zealand, Poland, Russian Federation, Spain, Switzerland, United Kingdom.	Tominersen	Intrathecally	Placebo	• The dataset pertains to the alteration observed in the Composite Unified HD Rating Scale Score-Z Score, with particular emphasis on the Total Motor Score Symbol Digit Modality Test and Stroop Word Reading. • Assesses the alteration in the Total Functional Capacity (TFC) Score, as measured at baseline and reported at Week 21 for the intervention group (ODC) and Week 69 for the control group (NDC).	Phase III
A Study of Treatment with Pridopidine (ACR16) in Participants with Huntington Disease	Austria, Belgium, France, Germany, Italy, Portugal, Spain, United Kingdom	ACR16	Not specified	Placebo	Week 26 Modified Motor Score (mMS) change from baseline. Dysarthria, tongue protrusion, finger taps (right and left), pronate/supinate hands, Luria—first hand-palm sequencing, arms rigidity, body bradykinesia, gait, tandem walking, and retropulsion pull test are mMS items.	Phase III
An Open-Label Extension Study to Evaluate Long-Term Safety and Tolerability of RO7234292 (RG6042) in Huntington disease Participants Who Participated in Prior Roche and Genentech Sponsored Studies	United States, Canada, Germany, Italy, Spain, United Kingdom.	Tominersen	Not specified	NA	Treatment-emergent adverse events are included in the 3-year data set after the last study drug intake. For safety, the Columbia Suicide Severity Rating Scale tracks composite endpoints over time to assess suicidal ideation and behavior. The Montreal Cognitive Assessment measure’s cognition with baseline and subsequent scores. All arms except Tominersen 120 mg every four weeks Q4W (Period 1) are in Milestone Period 2, with 3-year observations.	Phase III
Study of Memantine to Treat Huntington Disease	United States	Memantine	Typically administered orally	Placebo	Revision of Hopkins Verbal Learning Test for Delayed Recall. The Hopkins Verbal Learning Test-Revised has 3 pieces. Free recall ranges from 0 to 36, delayed recall from 0 to 12, and delayed recognition from -12 to 12. More points mean better function in all 3 parts. For each Hopkins Verbal Learning Test-Revised part, an average standardized z score is calculated. Subtracting baseline from later time point values yields change. For all alive patients missing post-baseline assessments, values were imputed. It measures cognitive function, specifically memory., Start of drug treatment, baseline, 3 months, 6 months	Phase IV
Alternatives for Reducing Chorea in Huntington Disease	United States, Australia, Canada	SD-809	Oral	Placebo	The number of participants who experienced treatment-emergent adverse events (TEAEs), serious TEAEs, severe TEAEs, drug-related TEAEs, and TEAEs leading to withdrawal during various treatment periods is reported in this data. Adverse events included any unfavorable medical occurrence in study drug participants, regardless of the possibility of a causal relationship. Severe AEs were defined as those that interfered with everyday activities.	Phase III

Table 2 summarizes HD clinical trials, focusing on treatments like Minocycline, deutetrabenazine, valbenazine, tominersen, pridopidine, and memantine. These studies show varied efficacy and safety outcomes. For instance, valbenazine improved chorea symptoms with a good safety profile, while minocycline and tominersen encountered high dropout rates due to adverse effects, highlighting challenges in long-term management. Pridopidine demonstrated potential benefits in motor functions, and memantine was well-tolerated, emphasizing the importance of developing tailored treatment approaches based on patient response and tolerability in clinical practice.

**Table 2:** Comparative outcomes of clinical trials investigating pharmacological treatments for Huntington disease.

Study title	Study parts	Dose regimens	Participants enrollment	Completion and dropout	Baseline characteristics	Adverse events	Principal conclusion
Pilot Study of Minocycline in Huntington Disease	The study involved initial screening, randomization, and follow-up phases.	Minocycline 100 mg Twice Daily, Matching Placebo Twice Daily.	134 patients were screened, and 114 were randomized at 12 Huntington Study Group clinical sites.	Started: Minocycline 87, Placebo 27; Completed: Minocycline 73, Placebo 22; Not Completed: Minocycline 14, Placebo 5	Total participants: 114; Age range: 18–65 years (96.5%), >65 years (3.5%); Mean Age (SD): Minocycline 47.1 (10.3), Placebo 47.8 (10.6); Gender: Female 53.5%, Male 46.5%	Serious Adverse Events: Minocycline 12.64%, Placebo 3.70%; Common adverse events include myocardial infarction, chest discomfort, pneumonia, and others.	High dropout rates and serious adverse events indicate significant challenges in the long-term management and tolerability of Minocycline.
First-Time Use of SD-809 in Huntington Disease	Screening, Randomization, Follow-up	SD-809 Tablets: Available in 6, 9, and 12 mg doses, all identical in size, shape, and color (White). SD-809 Placebo: Tablets identical in appearance to SD-809.	A total of 90 subjects were randomized 1:1 to receive either SD-809 or placebo.	Started: SD-809: 45, Placebo: 45; Completed: SD-809: 44, Placebo: 43; Not Completed: SD-809: 1, Placebo: 2	Total participants: 90; Mean Age: SD-809: 55.4 (SD 10.32), Placebo: 52.1 (SD 13.36); Sex: Female 44.4%, Male 55.6%; Race: Predominantly White 92.2%	Common adverse events include gastrointestinal disturbances and psychiatric disorders. Serious adverse events occurred at similar rates in both groups.	Treatment with SD-809 showed potential benefits in managing symptoms. Its safety profile is characterized by manageable adverse events, and high completion rates suggest acceptable tolerability.
Efficacy, Safety, and Tolerability of Valbenazine for the Treatment of Chorea Associated with Huntington Disease	Screening, Randomization, Follow-up, Maintenance Period	Valbenazine: Capsule, administered orally once daily for 12 weeks. Placebo: Identical capsules administered orally once daily for 12 weeks.	A total of 127 subjects were enrolled. 64 were randomized to Valbenazine and 63 to Placebo.	Started: Valbenazine: 64, Placebo: 64; Completed: Valbenazine: 57, Placebo: 54; Not Completed: Valbenazine: 7, Placebo: 10. Reasons for non-completion include withdrawals and study pauses.	Participants: 127; Mean Age: ~54 years; Gender: Female: 54.3%, Male: 45.7%; Ethnicity: Predominantly Non-Hispanic or Latino	Serious Adverse Events: Colon cancer and psychotic disorders in the placebo group, angioedema in the Valbenazine group. nonserious events include fatigue, falls, and somnolence.	Valbenazine showed a significant improvement in chorea symptoms compared to placebo. Both clinical and patient global impressions of change favored Valbenazine over placebo.
A Study to Evaluate the Efficacy and Safety of Intrathecally Administered RO7234292 (RG6042) in Participants with Manifest Huntington Disease	Original Design Cohorts (ODC) and New Design Cohorts (NDC)	Placebo Q4W (ODC & NDC), RO7234292 (tominersen) 120 mg Q4W (ODC), RO7234292 (tominersen) 120 mg Every 8 Weeks (Q8W) (ODC), Tomi 120 mg Q8W (NDC), Tomi 120 mg Q16W (NDC)	ODC: 36 per arm; NDC: 263 (Q8W), 264 (Q16W and Placebo)	Completed (ODC): 0 for all; Not Completed (ODC): 36 for all, reasons include adverse events, withdrawals, and lost to follow-up; Completed (NDC): 216 (Q8W), 207 (Q16W), 211 (Placebo); Not Completed (NDC): 47 (Q8W), 57 (Q16W), 53 (Placebo)	Mean Age: ~45–48 years across groups; Gender Distribution: ~46% female, 54% male overall; Ethnicity: Predominantly Not Hispanic or Latino	Higher adverse event rates in NDC (92.3–93.1%) compared to ODC (77.8–80%)	High dropout rates due to adverse events and withdrawals highlight the challenges in long-term clinical management of Huntington disease. Significant participant management and retention issues were noted, reflecting the complexity of administering chronic treatments.
A Study of Treatment with Pridopidine (ACR16) in Participants with Huntington Disease	Randomized Phase (26 Weeks), Open-Label Phase (26 Weeks)	Placebo/ACR16 90 mg: Randomized phase with placebo transitioning to ACR16. ACR16 45 mg/90 mg: Initial ACR16 45 mg, escalating to 90 mg. ACR16 90 mg/90 mg: Consistent ACR16 90 mg.	Total enrolled: 437 participants across 3 groups: Placebo/ACR16 90 mg, ACR16 45 mg/90 mg, ACR16 90 mg/90 mg.	Randomized Phase: Started: 437, Completed: 386, Not Completed: 51 (Adverse Events, Deaths, Withdrawals). Open-Label Phase: Started: 353, Completed: 305, Not Completed: 48.	Participants: 437; Mean Age: ~51 years; Gender: Female: 50.8%, Male: 49.2%; Predominantly Caucasian.	Adverse Events during the study include serious events like death and hospitalizations and nonserious events like fatigue and falls. Various side effects were reported per treatment group.	Significant motor function improvements in ACR16 groups compared to placebo. Extended treatment in the open-label phase showed sustained or improved outcomes in motor scores and functional assessments.
An Open-Label Extension Study to Evaluate Long-Term Safety and Tolerability of RO7234292 (RG6042) in Huntington disease Participants Who Participated in Prior Roche and Genentech Sponsored Studies	Period 1: All participants received Q4W dosing and transitioned to Period 2. Period 2: Participants were re-randomized to either Q8W or Q16W dosing.	Period 1: Tominersen 120 mg Q4W. Period 2: Tominersen 120 mg Q8W and Q16W.	Total enrolled: 236 participants across 3 arms. Initial 17 in Q4W, expanding to 138 in Q8W and 81 in Q16W in Period 2.	No participants completed the study. Reasons for not completing include the sponsor’s decision, adverse events, withdrawal by subject, death, and other specified reasons.	Total participants: 236; Age: Average ~48 years across periods; Gender: Mixed, with a slight majority male in Period 2; predominantly non-Hispanic or Latino.	High incidence of treatment-emergent adverse events across all dosing arms, with notable events including psychiatric disorders, gastrointestinal issues, and systemic disorders.	The study faced significant challenges with participant retention and adverse events, highlighting concerns over the tolerability and safety of the treatment regimen in a clinical setting.
Study of Memantine to Treat Huntington Disease	Period 1: Participants received either Memantine or Placebo. Period 2: Participants either continued on Memantine or switched from Placebo to Memantine.	Memantine Group: 10 mg twice daily for 6 months. Placebo, Then Memantine Group: Placebo for the first 3 months, then 10 mg Memantine twice daily for the next 3 months.	65 participants were screened; 6 failed screening; 9 declined to participate; 50 were randomized.	All 50 participants completed the study with no dropouts recorded.	Total participants: 50; Age: Mean 47.3 years; Gender: 31 females (62%), 19 males (38%). The standard deviation for age was not provided.	Minimal adverse events were reported, with only a few instances of diarrhea and dizziness in the Memantine group and 1 instance of dizziness in the placebo group.	Despite the study’s older data and some data loss over time, the trial demonstrated minimal serious adverse events and good retention across both arms, with notable switches from placebo to active treatment.
Alternatives for Reducing Chorea in Huntington Disease	Rollover Cohort: Participants from a previous study continued with SD-809 ER. Switch Cohort: Participants switched from tetrabenazine to SD-809 ER. Both cohorts had dose adjustments based on tolerability and effects.	Rollover Cohort: Started with 6 mg SD-809 ER, adjustable up to 72 mg/day, or 42 mg/day if on CYP2D6 inhibitors. Switch Cohort: Started with a dose equivalent to previous tetrabenazine therapy, adjustable up to 48 mg/day.	Rollover Cohort: 82 participants started, 56 completed. Switch Cohort: 37 started, 25 completed.	Total not completed: 38; Reasons include adverse events, protocol violations, and withdrawals by the participant or investigator.	Age average around 53 years; Gender: Rollover Cohort – 45% female, 55% male; Switch Cohort – 40.5% female, 59.5% male. Ethnicity mostly non-Hispanic or Latino.	Adverse events reported in both cohorts, including gastrointestinal issues, nervous system disorders, and other nonserious and serious events.	The study evaluated long-term use of SD-809 ER, showing feasibility of switching from tetrabenazine and continuing with SD-809 ER with a focus on managing chorea symptoms in Huntington disease.

## 4. Discussion

HD is an inherited neurodegenerative disorder that causing motor, cognitive, and psychiatric abnormalities. Currently, there is no curative treatment for HD. However, this paper provides valuable insights into the continuously evolving field of research, particularly the active investigation of innovative pharmaceutical interventions to improve symptom management and overall quality of life for individuals affected by HD. As of November 10, 2023, this study reveals a significant aggregate of 242 phase III and IV clinical trials for HD. However, only 8 trials were found to satisfy the specific inclusion criteria delineated in the study. Keywords utilized for the search included “Huntington disease,” “Huntingtin gene,” and “Chorea.”

Several limitations should be acknowledged in this study. Firstly, reliance solely on data from ClinicalTrials.gov may introduce bias, as it represents only 1 source of clinical trial information. Utilizing additional databases could provide a more comprehensive and global overview of clinical trials related to HD. Secondly, the completeness and quality of data extracted from ClinicalTrials.gov may vary, potentially impacting the reliability of findings. Incomplete or inaccurate data could influence the interpretation and generalizability of results. These limitations underscore the need for cautious interpretation and highlight opportunities for future research to address these challenges.

This study examines the diverse pharmacological characteristics of minocycline, specifically emphasizing its effects on microglial activation, inflammatory responses, and apoptotic pathways related to HD. This review also highlights the effectiveness and safety of other medications currently under investigation, such as deutetrabenazine, which serves as a component in the therapeutic management of chorea, a characteristic symptom observed in individuals with HD and tardive dyskinesia. The study also evaluates changes in the composite Unified HD Rating Scale Score-Z Score, providing significant insights into the motor and functional aspects related to HD. Additionally, pridopidine, a dopamine receptor modulator, has shown potential in alleviating motor deficits. Furthermore, the potential therapeutic effects of memantine, a medication commonly used in the treatment of Alzheimer disease, are currently being investigated in the context of alleviating symptoms associated with HD.

Table 2 summarizes HD clinical trials, focusing on treatments like minocycline, deutetrabenazine, valbenazine, tominersen, pridopidine, and memantine. These studies show varied efficacy and safety outcomes. For instance, valbenazine improved chorea symptoms with a good safety profile, while minocycline and tominersen encountered high dropout rates due to adverse effects, highlighting challenges in long-term management. Pridopidine demonstrated potential benefits in motor functions, and memantine was well-tolerated, emphasizing the importance of developing tailored treatment approaches based on patient response and tolerability in clinical practice.

### 4.1. Minocycline

Minocycline, belonging to tetracycline class of antibiotics, is a broad-spectrum antibiotic for managing diverse bacterial infections.^[28]^ The mechanism of action involves the inhibition of bacterial protein synthesis, leading to the slowing of the growth in both Gram-positive and Gram-negative bacteria. In addition to its antibiotic properties, minocycline has anti-inflammatory effects, suggesting it is a valuable therapeutic choice for managing inflammatory conditions.^[29]^ It has garnered attention in the context of HD due to its potential neuroprotective effects.^[30]^ Several studies have been conducted to examine the involvement of this medication in reducing neurodegenerative causes related to HD. These mechanisms include the attenuation of microglial activation and inflammatory reactions and the modulation of apoptotic pathways. Ongoing research is being conducted to explore the potential therapeutic impact of minocycline on neurodegenerative disorders such as HD due to its multifaceted pharmacological properties.^[31–33]^

### 4.2. Deutetrabenazine

Deutetrabenazine indicates a significant progression in the pharmacological treatment of chorea linked to HD and tardive dyskinesia. Deutetrabenazine is classified as a vesicular monoamine transporter 2 inhibitor.^[34]^ Its mode of action primarily involves modulating neurotransmitter release, specifically dopamine.^[35]^ Adopting a nuanced approach is of utmost importance when dealing with the involuntary and disruptive movements.^[36]^ Significantly, it was developed to serve as a more stable substitute for tetrabenazine, thereby providing the benefit of an extended duration of effectiveness and a decreased frequency of dosage.^[37]^ Deutetrabenazine exerts its therapeutic effects by modulating dopamine levels in distinct cerebral regions by inhibiting vesicular monoamine transporter 2, thereby facilitating the intensity of atypical motor manifestations linked to these neurologic conditions.^[38]^ Although it has demonstrated therapeutic advantages, it is not exempt from adverse effects; deutetrabenazine commonly includes drowsiness, insomnia, and fatigue.^[39]^

### 4.3. Tominersen

It is a pharmaceutical compound currently investigated by Roche and Ionis Pharmaceuticals for its potential therapeutic application in managing HD.^[40]^ It aims to mitigate the advancement of HD by diminishing the quantities of the mHtt protein.^[40]^ The safety and efficacy of tominersen in individuals with early manifest HD have been assessed through clinical trials. These trials aimed to evaluate the effects of this medication on multiple disease-related factors, encompassing both motor and cognitive function.^[41]^

### 4.4. Pridopidine

Pridopidine is a pharmaceutical compound presently undergoing examination for its prospective therapeutic utility in managing HD.^[42]^ Pridopidine acts as a dopamine regulator by modulating dopamine receptors’ activity, intending to augment the transmission of dopaminergic signals.^[43]^ Various clinical trials have been undertaken to evaluate the effectiveness of the medication in treating the motor and cognitive deficits linked to HD, yielding diverse outcomes.^[44]^ Regarding tolerability, pridopidine has demonstrated a favorable safety profile in clinical trials, with frequently observed adverse effects encompassing dizziness and incidents of falling.^[45]^

### 4.5. Memantine

Memantine, which is categorized as an N-methyl-D-aspartate (NMDA) receptor antagonist, represents a therapeutic alternative for individuals experiencing moderate to severe stages of Alzheimer disease.^[46]^ The precise mechanism by which it inhibits glutamate activity, a neurotransmitter involved in cognitive processes, facilitates the regulation of inter-neuronal communication within brain regions associated with memory and learning.^[47]^ Memantine is commonly administered orally and is frequently used in conjunction with cholinesterase inhibitors to effectively manage symptoms associated with Alzheimer disease and alleviate cognitive decline.^[48]^ Although it does not provide a cure, memantine demonstrates effectiveness in offering symptomatic relief and improving daily functioning for individuals who do not respond adequately to alternative medications for Alzheimer disease.^[49]^ In addition to its well-established use in Alzheimer disease, current research is investigating the potential of memantine in mitigating symptoms associated with HD.^[50]^ Despite being less well-established in the context of HD, recent research and clinical trials have shown that memantine is a topic of continued interest due to its potential ability to alleviate the cognitive and motor impairments associated with this genetic disorder.^[51]^

This review is an essential component in understanding the current therapy methods for these devastating neurodegenerative diseases. It identifies and analyses the potential efficacy and mechanisms of action of several medications, each presenting unique options for symptom therapy. The study emphasizes the importance of a comprehensive approach to illness treatment, acknowledging the supplementary role of non-pharmacological interventions such as physical and occupational therapy, speech therapy, counseling, support groups, and pharmacological treatments. Furthermore, it highlights the need for personalized treatment methods, acknowledging the variability of HD symptoms and the necessity for tailored medications to improve patient outcomes. The review provides a resource for focusing efforts to improve the quality of life for those affected by HD, with implications for future research and clinical practice.

## 5. Conclusion

Evaluating phase III and IV clinical studies on HD highlights the importance of tailored approaches for each patient’s unique disease presentation. Current medications aim to manage HD symptoms, potentially improving outcomes and reducing disease progression risks. Several key outcomes and the current status of the analyzed trials have been identified from the analyzed trials. Minocycline demonstrated a preliminary impact on HD progression but faced high dropout rates and serious adverse events, indicating challenges in long-term use. Deutetrabenazine showed potential benefits in managing symptoms with manageable adverse events and high completion rates, particularly effective in reducing chorea. This drug is now available for clinical use in managing HD symptoms. Valbenazine significantly improved chorea symptoms compared to placebo, with positive clinical and patient global impressions. It is currently used as a treatment option for chorea in HD patients. Pridopidine showed significant motor function improvements and sustained benefits in extended treatment, making it a valuable therapeutic option. Further studies are ongoing to confirm its long-term efficacy and safety. Memantine demonstrated minimal serious adverse events and potential cognitive benefits, specifically in memory, for HD patients. It is being further evaluated for its broader therapeutic potential in HD. These findings underscore the need for a comprehensive approach to HD treatment, focusing on personalized medicine and tailored strategies based on patient response and tolerability. Continued research is essential to enhance therapeutic options and improve the quality of life for individuals with HD. The data from ClinicalTrials.gov provide a foundational resource for advancing HD management. This review of phase III and IV trials offers a comprehensive look at the current state of HD treatment, emphasizing personalized strategies and highlighting areas for future research to develop more effective therapies.

## Acknowledgments

The author would like to acknowledge Deanship of Graduate Studies and Scientific Research, Taif University for funding this work.

## Author contributions

**Conceptualization:** Mohammed S Alharthi.

**Data curation:** Mohammed S Alharthi.

**Formal analysis:** Mohammed S Alharthi.

**Funding acquisition:** Mohammed S Alharthi.

**Investi**g**ation:** Mohammed S Alharthi.

**Methodology:** Mohammed S Alharthi.

**Project administration:** Mohammed S Alharthi.

**Software:** Mohammed S Alharthi.

**Validation:** Mohammed S Alharthi.

**Writing – original draft:** Mohammed S Alharthi.

**Writing – review & editing:** Mohammed S Alharthi.

## References

[1] Bano D Zanetti F Mende Y Nicotera P. Neurodegenerative processes in huntington’s disease. Cell Death Dis. 2011;2:e228–e228. https://www.nature.com/articles/cddis2011112. 22071633 10.1038/cddis.2011.112 PMC3223696

[2] McColgan P Tabrizi SJ. Huntington’s disease: a clinical review. Eur J Neurol. 2018;25:24–34. https://onlinelibrary.wiley.com/doi/10.1111/ene.13413. 28817209 10.1111/ene.13413

[3] Paoli R Botturi A Ciammola A. Neuropsychiatric burden in huntington’s disease. Brain Sci. 2017;7:67. http://www.mdpi.com/2076-3425/7/6/67. 28621715 10.3390/brainsci7060067 PMC5483640

[4] Raymond LA André VM Cepeda C Gladding CM Milnerwood AJ Levine MS. Pathophysiology of Huntington’s disease: time-dependent alterations in synaptic and receptor function. Neuroscience. 2011;198:252–73. https://linkinghub.elsevier.com/retrieve/pii/S0306452211009948. 21907762 10.1016/j.neuroscience.2011.08.052 PMC3221774

[5] Dalmau J Geis C Graus F. Autoantibodies to synaptic receptors and neuronal cell surface proteins in autoimmune diseases of the central nervous system. Physiol Rev. 2017;97:839–87. https://www.physiology.org/doi/10.1152/physrev.00010.2016. 28298428 10.1152/physrev.00010.2016 PMC5539405

[6] Obeso JA Rodriguez-Oroz MC Stamelou M Bhatia KP Burn DJ. The expanding universe of disorders of the basal ganglia. Lancet. 2014;384:523–31. https://linkinghub.elsevier.com/retrieve/pii/S0140673613624186. 24954674 10.1016/S0140-6736(13)62418-6

[7] Paulsen JS. Cognitive impairment in huntington disease: diagnosis and treatment. Curr Neurol Neurosci Rep. 2011;11:474–83. http://link.springer.com/10.1007/s11910-011-0215-x. 21861097 10.1007/s11910-011-0215-x PMC3628771

[8] Rosenblatt A Leroi I. Neuropsychiatry of HD and other basal ganglia disorders. Psychosomatics. 2000;41:24–30. https://linkinghub.elsevier.com/retrieve/pii/S0033318200711704. 10665265 10.1016/S0033-3182(00)71170-4

[9] Decruyenaere M Evers-Kiebooms G Boogaerts A. The complexity of reproductive decision-making in asymptomatic carriers of the huntington mutation. Eur J Hum Genet. 2007;15:453–62. https://www.nature.com/articles/5201774. 17245406 10.1038/sj.ejhg.5201774

[10] Cisbani G Cicchetti F. An in vitro perspective on the molecular mechanisms underlying mutant huntingtin protein toxicity. Cell Death Dis. 2012;3:e382–e382. https://www.nature.com/articles/cddis2012121. 22932724 10.1038/cddis.2012.121 PMC3434668

[11] Molero AE Arteaga-Bracho EE Chen CH. Selective expression of mutant huntingtin during development recapitulates characteristic features of huntington’s disease. Proc Natl Acad Sci USA. 2016;113:5736–41. https://pnas.org/doi/full/10.1073/pnas.1603871113. 27140644 10.1073/pnas.1603871113 PMC4878495

[12] Möller T. Neuroinflammation in huntington’s disease. J Neural Transm. 2010;117:1001–8. http://link.springer.com/10.1007/s00702-010-0430-7. 20535620 10.1007/s00702-010-0430-7

[13] Lee JM Ramos EM Lee JH. CAG repeat expansion in huntington disease determines age at onset in a fully dominant fashion. Neurology. 2012;78:690–5. https://www.neurology.org/doi/10.1212/WNL.0b013e318249f683. 22323755 10.1212/WNL.0b013e318249f683 PMC3306163

[14] Ross CA Aylward EH Wild EJ. Huntington disease: natural history, biomarkers and prospects for therapeutics. Nat Rev Neurol. 2014;10:204–16. https://www.nature.com/articles/nrneurol.2014.24. 24614516 10.1038/nrneurol.2014.24

[15] Nance MA. Therapy in huntington’s Disease: where are we? Curr Neurol Neurosci Rep. 2012;12:359–66. http://link.springer.com/10.1007/s11910-012-0277-4. 22544535 10.1007/s11910-012-0277-4

[16] Zarotti N Dale M Eccles FJR Simpson J. More than Just a brain disorder: a five-point manifesto for psychological care for people with huntington’s disease. J Pers Med. 2022;12:64. https://www.mdpi.com/2075-4426/12/1/64. 35055379 10.3390/jpm12010064 PMC8780585

[17] Dhingra H Gaidhane SA. Huntington’s disease: understanding its novel drugs and treatments. Cureus. 2023. https://www.cureus.com/articles/184798-huntingtons-disease-understanding-its-novel-drugs-and-treatments. 10.7759/cureus.47526 PMC10664735 38021751

[18] Asla MM Nawar AA Abdelsalam A. The efficacy and safety of pridopidine on treatment of patients with huntington’s disease: a systematic review and <scp>meta‐analysis</scp>. Mov Disord Clin Pract. 2022;9:20–30. https://movementdisorders.onlinelibrary.wiley.com/doi/10.1002/mdc3.13357. 35005061 10.1002/mdc3.13357 PMC8721839

[19] Frank S. Treatment of huntington’s disease. Neurotherapeutics. 2014;11:153–60. http://link.springer.com/10.1007/s13311-013-0244-z. 24366610 10.1007/s13311-013-0244-z PMC3899480

[20] Cepeda C Murphy KPS Parent M Levine MS. The role of dopamine in huntington’s disease. 2014;211:235–54. https://linkinghub.elsevier.com/retrieve/pii/B9780444634252000106. 10.1016/B978-0-444-63425-2.00010-6 PMC4409123 24968783

[21] Sepers MD Raymond LA. Mechanisms of synaptic dysfunction and excitotoxicity in huntington’s disease. Drug Discov Today. 2014;19:990–6. https://linkinghub.elsevier.com/retrieve/pii/S1359644614000580. 24603212 10.1016/j.drudis.2014.02.006

[22] Zadegan SA Kupcha L Patino J Rocha NP Teixeira AL Furr Stimming E. Obsessive-compulsive and perseverative behaviors in huntington’s disease. Behav Brain Res. 2024;458:114767. https://linkinghub.elsevier.com/retrieve/pii/S0166432823004850. 37984520 10.1016/j.bbr.2023.114767

[23] García-González X Cubo E Simón-Vicente L. Pharmacogenetics in the treatment of huntington’s disease: review and future perspectives. J Pers Med. 2023;13:385. https://www.mdpi.com/2075-4426/13/3/385. 36983567 10.3390/jpm13030385 PMC10056055

[24] Bonelli R Wenning G. Pharmacological management of huntingtons disease: an evidence- based review. Curr Pharm Des. 2006;12:2701–20. http://www.eurekaselect.com/openurl/content.php?genre=article&issn=1381-6128&volume=12&issue=21&spage=2701. 16842168 10.2174/138161206777698693

[25] Vaou O Frank S. Managing chorea in huntington’s disease. Neurodegenerative Dis Manage. 2011;1:295–306. https://www.futuremedicine.com/doi/10.2217/nmt.11.40.

[26] Niedzielska E Smaga I Gawlik M. Oxidative stress in neurodegenerative diseases. Mol Neurobiol. 2016;53:4094–125. http://link.springer.com/10.1007/s12035-015-9337-5. 26198567 10.1007/s12035-015-9337-5 PMC4937091

[27] Jurcau A Jurcau C. Mitochondria in huntington’s disease: implications in pathogenesis and mitochondrial-targeted therapeutic strategies. Neural Regen Res. 2023;18:1472. https://journals.lww.com/10.4103/1673-5374.360289. 36571344 10.4103/1673-5374.360289 PMC10075114

[28] Jonas, Murray and BAC. Minocycline. Ther Drug Monit. 1982;4:115–46. 7048646

[29] Asadi A Abdi M Kouhsari E. Minocycline, focus on mechanisms of resistance, antibacterial activity, and clinical effectiveness: back to the future. J Glob Antimicrob Resist. 2020;22:161–74. https://linkinghub.elsevier.com/retrieve/pii/S2213716520300230. 32061815 10.1016/j.jgar.2020.01.022

[30] Paldino E Balducci C La Vitola P. Neuroprotective effects of doxycycline in the r6/2 mouse model of huntington’s disease. Mol Neurobiol. 2020;57:1889–903. http://link.springer.com/10.1007/s12035-019-01847-8. 31879858 10.1007/s12035-019-01847-8 PMC7118056

[31] Kim HS Suh YH. Minocycline and neurodegenerative diseases. Behav Brain Res. 2009;196:168–79. https://linkinghub.elsevier.com/retrieve/pii/S0166432808005500. 18977395 10.1016/j.bbr.2008.09.040

[32] Bonelli RM Hödl AK Hofmann P Kapfhammer HP. Neuroprotection in huntington’s disease: a 2-year study on minocycline. Int Clin Psychopharmacol. 2004;19:337–42. 15486519 10.1097/00004850-200411000-00004

[33] Romero‐Miguel D Lamanna‐Rama N Casquero‐Veiga M Gómez‐Rangel V Desco M Soto‐Montenegro ML. Minocycline in neurodegenerative and psychiatric diseases: an update. Eur J Neurol. 2021;28:1056–81. https://onlinelibrary.wiley.com/doi/10.1111/ene.14642. 33180965 10.1111/ene.14642

[34] Claassen DO Philbin M Carroll B. Deutetrabenazine for tardive dyskinesia and chorea associated with huntington’s disease: a review of clinical trial data. Expert Opin Pharmacother. 2019;20:2209–21. https://www.tandfonline.com/doi/full/10.1080/14656566.2019.1674281. 31613641 10.1080/14656566.2019.1674281

[35] Richard A Frank S. Deutetrabenazine in the treatment of huntington’s disease. Neurodegener Dis Manag. 2019;9:31–7. https://www.futuremedicine.com/doi/10.2217/nmt-2018-0040. 30624137 10.2217/nmt-2018-0040

[36] Scarduzio M Hess EJ Standaert DG Eskow Jaunarajs KL. Striatal synaptic dysfunction in dystonia and levodopa-induced dyskinesia. Neurobiol Dis. 2022;166:105650. https://linkinghub.elsevier.com/retrieve/pii/S0969996122000419. 35139431 10.1016/j.nbd.2022.105650

[37] Dorfman BJ Jimenez-Shahed J. Deutetrabenazine for treatment of involuntary movements in patients with tardive dyskinesia. Expert Rev Neurother. 2021;21:9–20. https://www.tandfonline.com/doi/full/10.1080/14737175.2021.1848548. 33174440 10.1080/14737175.2021.1848548

[38] Kim A Lalonde K Truesdell A. New avenues for the treatment of huntington’s disease. Int J Mol Sci . 2021;22:8363. https://www.mdpi.com/1422-0067/22/16/8363. 34445070 10.3390/ijms22168363 PMC8394361

[39] Dean M Sung V. Review of deutetrabenazine: a novel treatment for chorea associated with huntington's disease. Drug Des Devel Ther. 2018;Volume 12:313–9. https://www.dovepress.com/review-of-deutetrabenazine-a-novel-treatment-for-chorea-associated-wit-peer-reviewed-article-DDDT. 10.2147/DDDT.S138828 PMC5818866 29497277

[40] Palpagama TH Kwakowsky A Faull R Waldvogel H Kennedy C Ferguson M. Current and possible future therapeutic options for huntington’s disease. J Cent Nerv Syst Dis. 2022;14:117957352210925. 10.1177/11795735221092517 PMC9125092 35615642

[41] Rocha NP. Clinical trials for huntington disease. Pract Neurol. 2020;69:69–76.

[42] Caron NS Dorsey ER Hayden MR. Therapeutic approaches to huntington disease: from the bench to the clinic. Nat Rev Drug Discov. 2018;17:729–50. https://www.nature.com/articles/nrd.2018.133. 30237454 10.1038/nrd.2018.133

[43] Waters S Ponten H Edling M Svanberg B Klamer D Waters N. The dopaminergic stabilizers pridopidine and ordopidine enhance cortico-striatal Arc gene expression. J Neural Transm (Vienna). 2014;121:1337–47. http://link.springer.com/10.1007/s00702-014-1231-1. 24817271 10.1007/s00702-014-1231-1

[44] Chen S Liang T Xue T Xue S Xue Q. Pridopidine for the improvement of motor function in patients with huntington’s disease: a systematic review and meta-analysis of randomized controlled trials. Front Neurol. 2021;12. https://www.frontiersin.org/articles/10.3389/fneur.2021.658123/full. 10.3389/fneur.2021.658123 PMC8159155 34054700

[45] Squitieri F Landwehrmeyer B Reilmann R. One-year safety and tolerability profile of pridopidine in patients with huntington disease. Neurology. 2013;80:1086–94. https://www.neurology.org/lookup/doi/10.1212/WNL.0b013e3182886965. 23446684 10.1212/WNL.0b013e3182886965

[46] Rogawski MA Wenk GL. The neuropharmacological basis for the use of memantine in the treatment of alzheimer’s disease. CNS Drug Rev. 2003;9:275–308. https://onlinelibrary.wiley.com/doi/10.1111/j.1527-3458.2003.tb00254.x. 14530799 10.1111/j.1527-3458.2003.tb00254.x PMC6741669

[47] Poddar MK Banerjee S Chakraborty A Dutta D. Metabolic disorder in alzheimer’s disease. Metab Brain Dis. 2021;36:781–813. https://link.springer.com/10.1007/s11011-021-00673-z. 33638805 10.1007/s11011-021-00673-z

[48] Dou KX Tan MS Tan CC. Comparative safety and effectiveness of cholinesterase inhibitors and memantine for alzheimer’s disease: a network meta-analysis of 41 randomized controlled trials. Alzheimers Res Ther. 2018;10:126. https://alzres.biomedcentral.com/articles/10.1186/s13195-018-0457-9. 30591071 10.1186/s13195-018-0457-9 PMC6309083

[49] Farlow MR Miller ML Pejovic V. Treatment options in alzheimer’s disease: maximizing benefit, managing expectations. Dement Geriatr Cogn Disord. 2008;25:408–22. https://www.karger.com/Article/FullText/122962. 18391487 10.1159/000122962

[50] Venuto CS McGarry A Ma Q Kieburtz K. Pharmacologic approaches to the treatment of huntington’s disease. Mov Disord. 2012;27:31–41. 21997232 10.1002/mds.23953

[51] Kumar A Kumar V Singh K. Therapeutic Advances for Huntington’s Disease. Brain Sci. 2020;10:43. https://www.mdpi.com/2076-3425/10/1/43. 31940909 10.3390/brainsci10010043 PMC7016861

